# Brain lateralization and neural plasticity for musical and cognitive abilities in an epileptic musician

**DOI:** 10.3389/fnhum.2013.00829

**Published:** 2013-12-06

**Authors:** Isabel Trujillo-Pozo, Isabel Martín-Monzón, Rafael Rodríguez-Romero

**Affiliations:** ^1^Laboratory of Psychobiology, Faculty of Psychology, Campus Santiago Ramón y Cajal, University of SevillaSevilla, Spain; ^2^Neuroradiology Unit, Radiodiagnostic Department, Virgen del Rocío University HospitalSevilla, Spain

**Keywords:** hemispheric lateralization, neuropsychology, temporal lobe epilepsy, wada test, memory, neural plasticity, Hippocampus and memory

## Abstract

The use of intracarotid propofol procedure (IPP) when assessing musical lateralization has not been reported in literature up to now. This procedure (similar to Wada Test) has provided the opportunity to investigate not only lateralization of language and memory functions on epileptic patients but also offers a functional mapping approach with superior spatial and temporal resolution to analyze the lateralization of musical abilities. Findings in literature suggest that musical training modifies functional and structural brain organization. We studied hemispheric lateralization in a professional musician, a 33 years old woman with refractory left medial temporal lobe (MTL) epilepsy (TLE). A longitudinal neuropsychological study was performed over a period of 21 months. Before epilepsy surgery, musical abilities, language and memory were tested during IPP by means of a novel and exhaustive neuropsychological battery focusing on the processing of music. We used a selection of stimuli to analyze listening, score reading, and tempo discrimination. Our results suggested that IPP is an excellent method to determine not only language, semantic, and episodic memory, but also musical dominance in a professional musician who may be candidate for epilepsy surgery. Neuropsychological testing revealed that right hemisphere's patient is involved in semantic and episodic musical memory processes, whereas her score reading and tempo processing require contribution from both hemispheres. At one-year follow-up, outcome was excellent with respect to seizures and professional skills, meanwhile cognitive abilities improved. These findings indicate that IPP helps to predict who might be at risk for postoperative musical, language, and memory deficits after epilepsy surgery. Our research suggests that musical expertise and epilepsy critically modifies long-term memory processes and induces brain structural and functional plasticity.

## Introduction

Neuroplasticity as a neural consequence of environmental enrichment or epileptic lesions has been previously reported in literature on human and comparative animal studies (Sutula, 2001, 2004; Elger et al., 2004; Rickard et al., 2005). Experimental studies in chronic animal models support the viewpoint that recurring alterations in synaptic transmission triggered by seizures evolve into morphological reorganization of neurons and neural circuits, especially in the hippocampus, which leads to functional deficits and enhanced seizure susceptibility (Stafstrom and Sutula, 2005).

Several findings related to humans have suggested significant interhemispheric speech and verbal memory processing reorganization occurring in left hemisphere epilepsies (Rausch and Walsh, 1984; Helmstaedter et al., 1997; Risse et al., 1997; Benbadis, 2001; Brázdil et al., 2003; Janszky et al., 2003; Rausch et al., 2003; Alessio et al., 2013). Previous structural and functional studies have demonstrated the effects of musical training on the brain. It has been documented that early musical training influences structural development, especially in the auditory and motor cortices (Münte et al., 2002; Rosenkranz et al., 2007; Hyde et al., 2009). In conjunction, music and epilepsy presents an ideal model for studying plasticity-dependent changes in cortical representation and laterality.

Neuroimaging studies have examined the neural bases of musical and semantic processing. Semantic memory relies mainly upon the middle, inferior temporal and inferior frontal gyrus (IFG) in the left hemisphere (Binder et al., 2009). Studies using musical stimuli have reported the involvement of the anterior part of the temporal lobes, either in the left hemisphere (Platel et al., 2003) or in both hemispheres, with activation of the middle part of the left superior temporal gyrus and the medial frontal cortices for recognition tasks (Satoh et al., 2006), and primarily the left IFG for familiarity tasks (Plailly et al., 2007). Moreover, functional magnetic resonance imaging (fMRI) studies on music and language syntax have shown neuroanatomical and functional parallels. In fMRI studies conducted during the processing of linguistic syntax (Kaan and Swaab, 2002; Vigneau et al., 2006, 2011) and musical syntax (Koelsch et al., 2002; Krumhansl, 2004; Koelsch et al., 2005b; Tillmann et al., 2003, 2006; Minati et al., 2008; Abrams et al., 2011), bilateral temporal and frontal activations (with different hemispheric weighting) have been frequently observed.

On the basis of previous studies, we have hypothesized that early musical training and epilepsy will have significant effects on the reorganization of musical, memory, and language functions in epileptic professional musicians. Considering the neurobiological substrates of epilepsy disease, it is clear that epilepsy could be viewed as an example of plasticity. Understanding functional plasticity in memory and in verbal and musical language networks associated with temporal lobe epilepsy (TLE) is central to foreseeing verbal and memory changes or musical decline following surgery. Intracarotid propofol procedure (IPP)—a Wada test version—offers interesting opportunities to investigate the effects of epilepsy on the performance of these cognitive abilities, and to draw hypotheses about the cerebral representation of various aspects of these cognitive functions.

In this study, we propose introducing a novel neuropsychological battery to be used during IPP. It offers functional mapping approaches with superior spatial and temporal resolution (Wada and Rasmussen, 1960; Lüders et al., 1986). This procedure shows the persistence or loss of a function (speech, music or memory) during inactivation of one hemisphere or the other. Thus, the use of gold-standard clinical testing methodologies can increase the understanding of cognitive functions such as musical skills.

The aim of the current study was to investigate the interhemispheric reorganization of the musical, language, and memory functions in a professional musician with left TLE using IPP. We examined for the first time memory, language, and musical cognition in a professional musician before and after epilepsy surgery. Only one previous study has analyzed musical cognition after surgery (Schultz et al., 2005). Furthermore, the use of an IPP to study musical lateralization has not been previously reported in literature.

A second issue addressed in the present study concerns possible functional changes in cognitive functions after undergoing selective amygdalohippocampectomy (SAH) in this single case. A longitudinal study where we can evaluate cognitive changes in language, memory, and musical self-awareness deficits in a professional musician is highly elaborated and exceeds the scope of traditional neuropsychological evaluation.

The overall data supports our decision to conduct an exhaustive analysis of musical, memory, and linguistic processing prior to surgery in patients with medial temporal lobe (MTL) lesions who are professional musicians.

## Materials and methods

### Subject and case history

A 33-year-old right-handed woman, a professional pianist, and music teacher, who suffered from refractory left medial TLE. She started her training as a musician when she was 6, and after 3 years began to play piano. She sought treatment for possible surgical relief of refractory epilepsy. The patient provided consent, and the local ethics committee approved the study protocol. The suspected seizure focus was localized according to standard pre-surgical evaluation protocol on the basis of all available data including video- electroencephalography (EEG), neurological examination, structural Magnetic Resonance Imaging (MRI), neuropsychological testing, and Single Photon Emission Computed Tomography (SPECT). Because the neuropsychological results contradicted neuroimaging data, the patient was selected for IPP to evaluate not only cerebral language and memory dominance, but also the lateralization of cognitive musical abilities. The patient's characteristics and a summary of diagnosis appear in Table 1.

**Table 1:** **General epilepsy history and summary of diagnosis**.

**Sex**	**Woman**
Age	33 years
**EPILEPSY**	
Brain insult	TBI when she was 1 year old
Febrile seizure	No
First epileptic seizure	9 years old
Age of onset Auras	6 years old
Duration of seizures	24 years
Frecuency	On *cluster/4–5* month
Complex partial seizures	Yes
Antiepileptic treatment	Trileptal, Keppra, Noiafren and Stilnox
Neurological examination	Normal
Ictal EEG	Video EEG predominance interictal activity in left temporal lobe
Interictal EEG	Video EEG ictal activity in left temporal lobe
MRI	Reduction of left hippocampal size, with hypointensity and loss of internal differentiation
Interictal SPECT HMpao	Left anterior temporal lobe hypoperfusion
**SURGERY**	
Surgery procedure	Selective amigdalohippocampectomy transilvian (Yasargil technique)
Size of resection	Resection of the hippocampal gyrus, the subiculum and the parahippocampal gyrus. Resection of the anterior portion of the left hippocampus with preservation of the dorsal portion and tail
Histology	Hippocampal sclerosis. Massive pyramidal cells loss in Ammon's sectors (CA1 to CA4), astrocytosis and neuronal dispersion of dentate gyrus

### Background assessment: general neuropsychology, neuroimaging, electrophysiology, audiometry and music perception

Use of intracarotid propofol is a feasible and reliable method to determine the risk that surgery poses to anterograde memory and to determine which hemisphere is eloquent for language. Our study included an innovative test to assess the lateralization of musical abilities during IPP.

In this research a longitudinal neuropsychological study was conducted in 3 phases (9 months pre-surgery, 6 months post-surgery, and 12 months post-surgery).

Longitudinal musical assessment employed a musical questionnaire and series of musical tasks, named “Sevilla Battery for Evaluation of Musical Lateralization in order to assess music perception, recognition, learning, and memory cognitive processes. This research also involved exhaustive neuropsychological, neuroimaging, and neurological analysis.

#### Pre- and post-neuropsychological assessment

The patient's cognitive abilities were assessed using a series of standardized neuropsychological tests (Table 2).

**Table 2:** **Test scores obtained after neuropsychological assessment 9 months pre-surgery (Score I), 6 months (Score II) and 12 months (Score III) post-surgery**.

**Cognitive function**	**Test**	**Score I**	**Score II**	**Score III**
**GENERAL INTELLECTUAL FUNCTION**				
Intelligence scale (WAIS-III)	VerballQ	99	103	110
	Performance IQ	98	114	118
	Full scale IQ	99	108	114
**ATTENTION AND WORKING MEMORY**				
Attention/concentration	Trail making test A	54	34	29
	Letter cancellation (in seconds)	103	82	105
	Digit symbol-coding (WAIS III)	56	65	75
Selective attention/working memory	Digit span backwards (WAIS-III)	10	9	10
	Letter number sequencing (WAIS III)	13	11	13
	Arithmetic (WAIS III)	8	11	12
**SPAN MEMORY**				
	Digit span forwards (WAIS III)	13	8	10
**VISUAL MEMORY**				
	Rey complex figure			
	Inmediate recall	14, 5	19	21, 5
	Delayed recall	14, 5	17, 5	21, 5
	Design (WMS-III)			
	Inmediate recall	97	99	104
	Delayed recall	44	90	104
**SEMANTIC MEMORY-LANGUAGE**				
	Boston naming test	65	50	54
	Similarities (WAIS-III)	22	26	29
	Vocabulary (WAIS-III)	42	45	45
**VERBAL MEMORY**				
Stories recall	Logical memory (WMS-III)			
	Inmediate recall	25	35	35
	Delayed recall	16	22	23
Learning and memory words list	Selective reminding test			
	Inmediate recall	114	104	115
	Delayed recall	12	7	11
**EXECUTIVE FUNCTION**				
	Matrix reasoning (WAIS-III)	23	24	22
	Trail making test B	–	77	54
	STROOP colors and words	3.3	3.15	−3.91
	Verbal Fluency			
	Phonetic	15/13/2	12/13/5	9/8/9
	Category	17	12	14
	Wisconsin card sorting test			
	Categories	6	6	6
	Perseverative errors	5	8	5
**VISUOSPACIAL FUNCTION**				
	Benton visual form	14	16	16
	Stories (WAIS-III)	16	18	16
	Picture completion (WAIS-III)	20	24	24
**VISUOCONSTRUCTION**				
	Rey complex figure copy	35	36	36
	Block design (WAIS-III)	47	46	55

Pre-surgical neuropsychological assessment showed intelligence quotient (IQ) above average. The results also showed impairments in learning and memory cognitive processes using verbal and visual material. Assessment of executive skills showed preservation of set shifting and reasoning abilities, and no sensitivity to interference (see Table 2).

#### Neuroimaging

MR images were obtained with a 1.5 T Philips scanner (Eindhoven, Netherland). Axial and coronal 3D T1 Morphological MRI sequences, inversion recovery, axial, and coronal T2, and fluid-attenuated inversion recovery (FLAIR), were implemented for an optimal study, following an exhaustive pre-surgical epilepsy evaluation protocol. Interictal Tc-HMPAO brain SPECT was performed to locate seizure focus.

**Figure 1:**
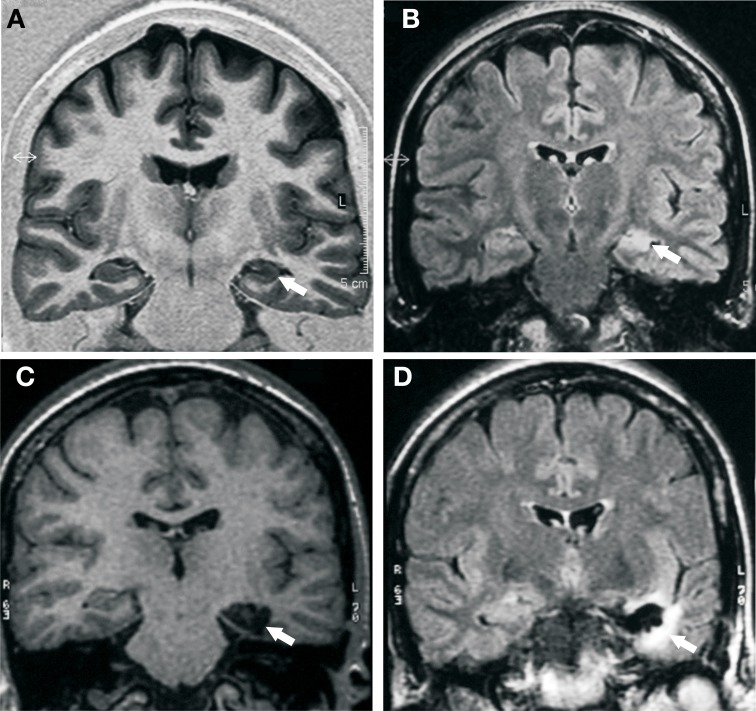
**Pre- and post-surgical MRI study. (A)** Pre-surgical MRI; IR coronal image shows reduction of left hippocampal size, with hypointensity, and loss of internal differentiation (white arrow). (**B**) Pre-surgical MRI; Flair coronal image reveals hyperintensity and decreased hippocampal size (white arrow). **(C)** Post-surgical MRI; T1 coronal image shows the absence of the hippocampal gyrus, the subiculum, and the parahippocampal gyrus, medial to collateral sulcus (white arrow). **(D)** Post-surgical MRI; Flair coronal image hyperintensity is observed, surrounding the surgical cavity, secondary to gliosis (white arrow).

A detailed MRI study was performed pre- and post-surgery for left transylvian SAH (Figure 1).

#### Electrophysiology

In the pre-surgery phase, the patient was evaluated with prolonged video-EEG-monitoring. The continuum scalp record showed pre-dominant interictal and ictal activity in the left temporal lobe.

#### Audiometry and music perception

Conventional (250–8000 Hz) pure tone audiometry was performed on the patient through the use of an Inter-Acoustic AC 40 diagnostic audiometer. The criteria used to define normal hearing, was that of pure tone thresholds of 25 dBHL or lower across all frequencies, with the absence of an air-bone gap (Martin and Clark, 2003). The audiometric assessment revealed pure tone audiometry and otoacoustic emissions within normal limits for her age (20–25 db).

Musical perceptual abilities were assessed using the Montreal Battery for Evaluation of Amusia (Peretz et al., 2003). The musician was asked before and after surgery with respect to special musical skills like melody processing, musical memory, rhythm, meter, harmony/dissonance, timbre, concentration and endurance, emotionality, and absolute pitch. The patient reported no music problems in her professional background.

### Experimental assessment of music processing and brain lateralization effects during intracarotid propofol procedure: stimuli and general procedure

#### Stimuli

Music involves complex brain functions underlying acoustic analysis, auditory memory, auditory scene analysis, and processing of musical syntax and semantics. We designed the “Sevilla Battery for Evaluation of Musical Lateralization” to assess the contribution of each brain hemisphere to music perception, recognition, learning, and memory cognitive processing. These tasks were used during IPP in the same order after left and right injection. The first one task performed was melody recognition task, followed by score-reading task, and tempo task. Two experimental conditions were presented in this study, the encoding and retrieval conditions. The encoding condition was performed under IPP, whereas the retrieval condition was performed after anesthetic recovery. In the encoding condition, the aim was to present different musical stimuli in order to explore musical semantic knowledge, musical reading, and tempo discrimination. In the retrieval condition, the aim was to assess free recall and recognition of musical stimuli presented in encoding condition. Two alternate forms of the musical test containing stimuli in the same order. At the beginning of IPP, before encoding stimuli, the subject was told that she would perform retrieval task later. The patient was tested on the following three musical tasks.

#### Melody recognition task

Musical excerpts from classical melodies were selected prior the carrying out of the IPP in order to decide the appropriate stimuli. Our decision was based on the responses marked by the patient during the musical questionnaire. Each one of the musical excerpts titles used as stimuli during the present task was known by the patient. During encoding condition, we presented a musical excerpt from “Tchaikovsky's Piano Concerto” and another from “The Marriage of Figaro” by W. A. Mozart” in order to assess left and right hemisphere functions, respectively. During retrieval condition, we evaluated free recall: the patient had to recall the title of the melody heard at the encoding condition. Later we assessed recognition: the patient had to listen three different melodies and was asked to choose which was the melody heard in the encoding condition. These stimuli were presented in random order for assessing left and right hemisphere. Each musical stimulus was presented for 9 s with inter-stimuli interval of 6 s.

#### Score-reading task

**Figure 2:**
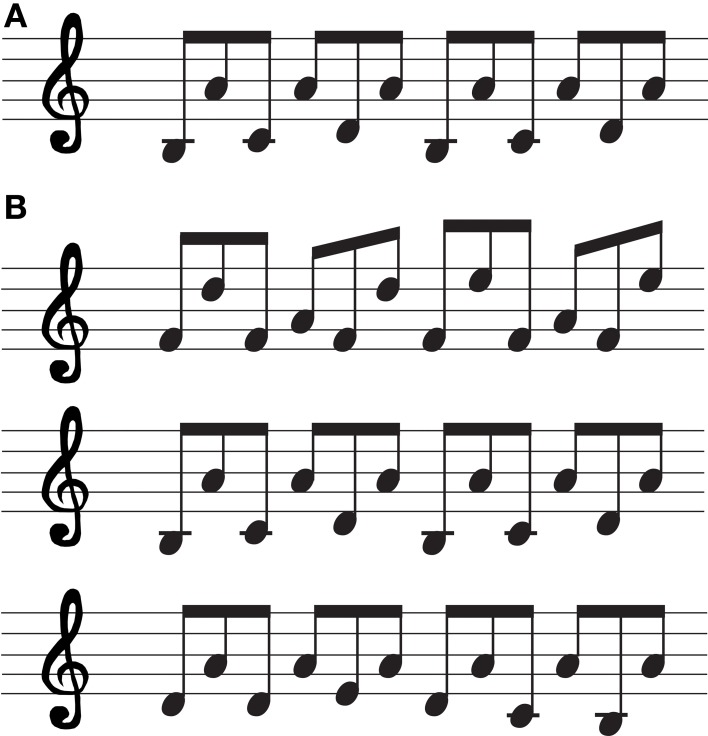
**Score-reading task. (A)** Musical score excerpt presented during encoding condition (left hemisphere inactivated). **(B)** Musical scores excerpts presented during retrieval condition (in order to assess left hemisphere functions).

Short musical piano scores from classical melodies were selected to be read aloud during IPP. Reading aloud meant that the patient could keep the rhythm and dynamics, but the pitch of the melody was not maintained. Reading aloud a score and singing it are different issues, since we can appreciate that is not the same listening to an opera sung than reading it aloud. During encoding condition, the patient was asked to read an excerpt of a score. We presented a different musical score excerpt in order to assess each hemisphere. They were located in an optimum angle, just in front of patient's eyes, ipsilateral to brain anesthetic injection, to guarantee the score-reading. During retrieval condition, in free recall, the patient had to spontaneously generate the previously read score. For recognition, the patient had to read three different music scores of similar difficulty and had to choose which one was the music score read during encoding condition (Figure 2).

The three different musical scores were presented on a sheet of paper simultaneously, to be read by the patient so she could recognize it. Each musical stimulus was presented for 10 s.

#### Tempo task

Tempo discrimination was assessed during IPP. During encoding condition, the patient was asked to pay attention of a musical excerpt in which only tempo modifications were introduced in the initial melody (14 s), thus creating a musical excerpt melody 7 s faster than the original melody (Chopin's Prelude Op 28 n° 4) and another one 17 s slower than the original melody (Debussy's Arabesque n°. 1) in order to assess left and right hemisphere functions, respectively. A melodic theme was chosen from one of the melodies unfamiliar to the patient according to her answers after the administration of the questionnaire. During retrieval condition, we presented three musical excerpts with fast tempo, normal tempo, and slow tempo, respectively; one of them was previously presented in the encoding condition. The musical excerpts were displayed in a random order to assess each hemisphere function. The patient had to recognize which one of the musical excerpts had been listened before and had to try to recognize it. The inter-stimuli interval was 6 s. Each auditory item was presented binaurally to the patient through an HP Pavilion Entertainment computer. All this data were stored on a special compact disc.

### Procedure

#### Intracarotid propofol procedure

Carotid catheterization was performed using a transfemoral approach, and cerebral angiography was performed prior to the IPP. It was performed to ascertain whether there were any anomalous circulation patterns that might influence the cerebral distribution of the anesthetic propofol. After selective catheterization of each internal carotid artery, posteroanterior, and lateral view was performed to determine whether crossover was present. Propofol was manually injected via a catheter into the internal carotid artery 1 cm distal to the bifurcation using a transfemoral approach. The side ipsilateral to the side of the suspected seizure focus (left hemisphere) was injected first.

During this procedure, 10 mg of propofol was injected into each one of the carotid arteries. Both right and left administration were conducted on the same day with a delay of 35 min between injections. The patient was evaluated on the motor strength of the upper extremities by manual muscle testing according to the Medical Research Council. Patient motor strength was estimated by the recovery time to motor Grade 3 (T3/5) and Grade 5 (T5/5) after the injection. During IPP, bioelectrical brain activity was recorded by video-EEG. The complete procedure lasted 75–80 min.

#### Encoding condition

Immediately on occurrence of hemiplegia, with the effects of the drug apparent (during periods T3/5-T0/5-T3/5), the encoding condition of IPP was initiated. Firstly, hemispheric language dominance was assessed by the presence or absence of paraphasia, speech arrest, naming, and comprehension of simple commands. Secondly, the musical protocol was initiated. Three sets of musical memory items were presented. They included one classical melody shown for listening, one excerpt of piano score for oral reading, and a melody synthesized by a professional pianist with a minimal tempo modification. Finally, hemispheric verbal and visual memory performance was assessed by presenting the patient with four sets of items. They included four written words, four real objects, four line drawings of simple objects, four line drawings of abstract figures, and one simple sentence. Stimuli were presented at an average interval of 5 s. Appropriate perception of and focusing on the stimuli were controlled by gently moving the card in the patient's central visual field and the visual field ipsilateral to injection and observing corresponding saccadic eye movements. An identical procedure with another set of stimuli was used to test the other hemisphere immediately on placement of the catheter in the other hemisphere. The neurologist validated the anesthetic periods.

#### Retrieval condition

Retrieval condition began after the level of patient's consciousness and motor strength returned to baseline 25 min after the injection. Free recall and choice-recognition verbal, visual, and musical memory were tested.

#### Language, verbal and visual memory assessment

Language and memory were assessed during encoding and retrieval conditions of IPP. Language processing was tested first by means of comprehension and speech functions. We assessed comprehension by asking the patient to follow some simple commands. We assessed speech by asking the patient to verbalize some basic information. Speech was further analyzed by presenting an object to be named. Hereafter, memory processing was tested using seventeen memory items. These memorization stimuli consisted of four black and white concrete objects pictures; four abstracts words, a simple sentence, four line drawings, and four common objects. The patient was then left to rest, supported by a specialist epilepsy nurse and monitored by medical staff. For baseline score and each hemisphere injection, memory accuracy was assessed for each stimulus type using a measure of discrimination. We computed the memory scores by subtracting the baseline discrimination score from each injection's discrimination score and dividing the difference by the total number of possible targets. We grouped verbal targets (four abstracts words and a sentence), visual targets (four line drawing pictures), and doubly encoding targets (four black and white concrete objects pictures and four common objects). Our results were ranged between 0 and −1. Score 0 was memory totally lateralized and −1 no memory reserve. This scoring method was selected because it accounts baseline performance and the score for each injection. To our knowledge, only the studies by Kelley et al. (2002) and Testa et al. (2008) incorporated baseline performance into their hemispheric memory accuracy in Wada test analyses.

#### Musical assessment

During this period, free recall and recognition paradigms (identification of the target stimulus among an array of three stimuli) were employed as described above. We first asked the patient to remember the classical melody previously heard during encoding condition, and then to recognize this item by multiple choices with two other melodies heard. Next the patient was asked to remember the excerpt of a piano score that she read during encoding condition; then she was asked to recognize this item through multiple choices with two other options. Finally, we asked the patient to listen to a musical excerpt with timing modifications and she was asked to recognize it using multiple choices with other two options.

IPP memory scores were computed for the patient: percentage of musical memory items recognized on the first ipsilateral injection (i.e., the contralateral musical memory score), and percentage of musical memory items recognized on the second contralateral injection (i.e., the ipsilateral musical memory score).

## Results

### Pre- and post-neuropsychological assessment

The general history of the epilepsy and the results of diagnostic testing pre- and post-surgery are summarized in Tables 1, 2. Pre-surgical neuropsychological testing showed deficits in attention, as well as in visual and verbal short and long-term memory. Using the Yasargil-SAH technique (Yaşargil et al., 1985), the results showed a significant improvement over time. Post-surgical neuropsychological assessment showed improvement of at least one standard deviation (*SD*) in these cognitive functions at 6 months and 1 year post-surgery.

Specifically, at the 6-month follow-up post-surgical assessment, results showed impressive visual memory improvement in the Design subtest (WMS-III) and above one *SD* in the Rey–Osterreith Complex Figure Test. Verbal memory improvement presented a similar pattern in the Logical Memory subtest (WMS-III). Normal rates of execution were found for tests of attention/concentration such as Letter Cancellation Test, Trail Making Test A (TMTA), Trail Making Test B (TMTB) and Digit Symbol. These post-operative changes were consolidated over the first year (Table 2: Score III). At the 12-month follow-up post-surgical assessment, results showed a significant improvement in verbal IQ and performance IQ. Our longitudinal results showed an improvement cognitive pattern.

Post-surgical testing of the musician's self-assessment of her musical abilities with respect to memory, concentration, learning, endurance, apperception of absolute pitch, harmony, dissonance, timbre, melody, rhythm, meter, emotion, and interaction with others in making music revealed the following: improved concentration, learning, timbre, meter, rhythm, emotionality, and adaptation in making music with others.

We evaluated the patient's performance in score-reading, melody recognition and tempo discrimination pre- and post-surgery. The patient's results showed a perfect performance in each musical task. Memory performance using musical stimuli was not affected. Significant improvements in mood and quality of life were reported.

### Surgery outcome and histopathology

Pre-surgical MRI and SPECT studies revealed left hippocampal sclerosis, and hypoperfusion of the anterior temporal lobe, respectively. No others structures showed any affectation. After surgery, neuroimaging, and histopathology results were analyzed in detail. The patient underwent a left SAH, where the anterior portion of the left hippocampus, amygdala, parahippocampal gyrus, and subiculum were removed (Figures 1C, D). Post-surgical MRI (12 months post-surgery) showed in T1 coronal image absence of the left hippocampal gyrus, the subiculum, and the parahippocampal gyrus, medial to the collateral sulcus. In Flair coronal image, hyperintensity was observed surrounding the surgical cavity, secondary to gliosis. MRI showed the limit of the surgery resection, just until the collateral sulcus angle. Heschl's gyrus was intact and patient was reported to have normal hearing after surgery.

Neurologically, our patient has been free of all episodes for more than 12 months; she was classified as seizure free. This agrees with Engel's outcome classification Class 1 A (Engel et al., 1993).

EEG activity carried out 12 months post-surgery did not show abnormal epileptiform pattern.

Neuropathological examination of the resected hippocampus revealed massive loss of hippocampal pyramidal neurons of the four sectors of Ammon's horn (CA1–CA4), discrete reactive astrocytosis, and neuronal dispersion enlargement of the dentate gyrus. In the adjacent entorhinal cortex we observed a disorganization of cortical lamination, presence of ectopic neurons in white matter, hyalinization of the vessel wall and perivascular amylaceous bodies.

### Intracarotid propofol procedure results

#### Neuroradiological and anesthetic results

After a standard cerebral angiography on each hemisphere via the right femoral artery, no pathological results appeared. During anesthetic registration no complications were noted immediately during or after the procedure in each hemisphere.

In the left hemisphere, clinical assessment showed maximum anesthetic effect after 4 s (T3: 4 min; 30 s; T5: 5 min; 15 s) and in the right hemisphere after 3 s (T3: 3 min; 31 s; T5: 4 min; 56 s). We presented all verbal and musical stimuli as long as anesthetic effect was active in T0–T3.

#### Video-EEG register

After the propofol injection, the function of each hemisphere was monitored by EEG. EEG after propofol injection usually showed high delta activity in the injected hemisphere with frequent slowing, to a lesser degree, in the non-injected side, followed by a gradual disappearance of delta activity. The appearance of slow waves is frequently used to estimate the duration of drug effect, but the significance of slowing in the contralateral hemisphere after injection has not been well established.

**Figure 3:**
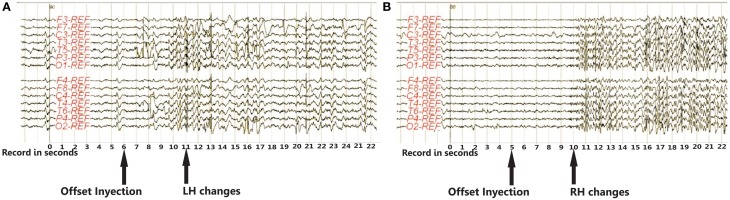
**EEG activity recorded from 6 s. after propofol injection in the left hemisphere (A) and from 5 s. in the right hemisphere (B).** Both EEG recordings show bilateral theta-delta slowing and theta band activity peaks post injection, which gradually decrease. No significant inter-hemisphere asymmetry is revealed. EEG activity returns to pre-injection baseline status approximately 10 min. after propofol injection. Abbreviations: LH, left hemisphere; RH, right hemisphere.

In this patient the changes in EEG activity were recorded from 6 s after propofol injection in the left hemisphere and in EEG activity from 5 s after propofol injection in the right hemisphere (Figure 3).

Both EEG records show bilateral theta-delta slowing, and theta band activity peaks in the first minute post-injection, decreasing gradually thereafter. No significant inter-hemisphere asymmetry was revealed. EEG activity returns to pre-injection baseline status approximately 10 min after propofol injection.

#### Physiological response

The physiological responses recorded after propofol injection in the left hemisphere were right hemianopsia, right central facial, and left gaze deviation; meanwhile in the right hemisphere they were left facial central, left hemianopsia, left facial central, right gaze deviation, jaw closure, and dystonic posture in left upper limb.

#### Language, verbal and visual memory results

Language function was located in the left hemisphere. Patient could not repeat, name objects or follow simple commands after the application of anesthetic to that brain hemisphere. The patient recovered language functions at 6 min 10 s.

Memory processing was mainly detected in right hemisphere. Memory performance was better after left hemisphere anesthesia than for the right hemisphere, showing a major effect in material recognition. In general, results from visual memory tests scores for the right hemisphere were higher than those of the memory verbal tests.

Memory lateralization was assessed by the number of items recognized. The memory score for left hemisphere injection was −0.4 for verbal stimuli, −0.25 for visual stimuli and 0 for double-encoded stimuli. In right hemisphere memory injection scores were −0.4 for verbal stimuli, −0.4 for visual stimuli and −0.5 for double encoded stimuli. These results showed that recognition of real objects and black and white concrete pictures was worse after injection of the “healthy” hemisphere than it was after injection of the hemisphere of seizures focus. Verbal stimuli results suggest that these stimuli are processed by both hemispheres and memory can be disrupted by dysfunction in either hemisphere. However, recognition of visual stimuli was better after injection into the left hemisphere. The memory score was −0.25, indicating right hemisphere lateralization for visual stimuli. As expected, memory was much less accurate after anesthetization of the hemisphere contralateral to the seizure focus. A material injection side interaction revealed that memory was worse for real objects after right than left injection. There was no difference between verbal stimuli recognition in both hemispheres, indicating no hemispheric verbal memory lateralization; this result suggested bilateralization for this material.

This suggests that the left and right hemispheres are critical for recognition of words and sentences. Conversely, memory for visual stimuli was better in left injection than in right injection. This showed impairment in the functioning of the left hemisphere, either by a temporal lobe seizure focus or propofol, resulting in impaired verbal memory.

#### Musical results

***Left hemisphere vs. right hemisphere***. Musical results after anesthetic to left and right hemisphere indicated different roles of these areas in music processing. Our findings in melody recognition task show that the patient could not remember the precise title of the musical excerpt stimulus (familiar melody) when the right hemisphere was anesthetized, meaning that this hemisphere is involved in music knowledge, and music memory process. Right-hemisphere regions are involved in the retrieval of melodic traces in perceptual memory; whenever the left hemisphere was anesthetized she remembered the precise title of the musical stimulus (familiar melody) by free recall and recognition.

The results in score-reading task, when the left hemisphere was anesthetized, indicated that the patient only could remember the notes or the iconic representation, but she couldn't free recall, whereas she could visually recognize the music score shown. Besides, we should underline that the patient was not able to read the score in encoding condition, since she was aphasic. After right hemisphere injection, during encoding condition when the right hemisphere was anesthetized, the patient read the music score shown perfectly despite of not singing it; however, during retrieval condition she couldn't remember it. This result was highly interesting because although music syntactic processing (reading) was lateralized in the left hemisphere, singing abilities involved contralateral brain structures. When the patient was asked to recognize and to choose which one of the three different music scores was presented during encoding condition, she could not perform it correctly. Therefore, this result in memory musical assessment suggests that syntactic aspects of music are pre-dominantly lateralized in the left hemisphere.

The results obtained for the tempo task were significantly different from the previous task. Results after anesthetization of the left and right hemisphere were found to be identical. During the retrieval condition, results show that the patient could not recognize which melody was heard on the encoding condition. This indicates that there is no hemispheric pre-dominance for identifying changes in tempo for melodies.

Our data in IPP revealed, with different musical tasks, how semantic and episodic musical memory functions showed a different pattern of brain activity lateralization.

Propofol injection and musical neuropsychological assessment led to the conclusion of performing a transylvian selective amigdalohippocampectomy to preserve musical functions in this patient.

## Discussion

Early musical training and epilepsy have strong effects on reorganization of musical and cognitive abilities in epileptic musicians. Musical, memory, and language lateralization is of critical importance in the evaluation for resective epilepsy surgery. In this study there are three major results. Firstly, we demonstrated that IPP is an excellent method to determine musical and language dominance, as well as semantic and episodic memory capacity in TLE surgery candidates. Secondly, we demonstrated that visual and verbal memory outcomes after left SAH presented an appropriate option vs. anterior temporal lobe resection (ATLR). We showed that memory results in IPP were a successful indicative of post-operative verbal and visual memory changes and music decline compared to other related factors such as language lateralization, preoperative performance on neuropsychological tasks and duration of epilepsy. Finally, our longitudinal analysis showed changes in cognitive functions, allowing a consideration of clinically significant post-operative plasticity in individual patients. We attempted to define functional anatomy of episodic and semantic memory for music, besides language, and memory lateralization. According to the available literature, our results demonstrate an overlap between many music-related functions and language processing (Platel et al., 1997; Maess et al., 2001; Koelsch et al., 2002, 2005a,b, 2006; Patel, 2003). Both abilities share fundamental characteristics such as syntactic and semantic structure, and written representations (Patel et al., 1998; Patel, 2003; Koelsch et al., 2004, 2005a,b, 2006). In order to analyze these functions we designed a battery named “Sevilla Battery for Evaluation of Musical Lateralization,” that involved listening, reading melodies, and tempo discrimination.

Our findings demonstrate reliable contributions between the two cerebral hemispheres for semantic, episodic, and syntactic processing of different types of materials.

The melody recognition task involved both hemispheres since the patient recognized the musical excerpt after selective inactivation of left and right hemispheres, but only could remember it when the right hemisphere was active. This IPP task provided us valuable information about neural organization that normally underlies various musical cognitive functions. Contrasting with the large number of functional neuroimaging studies that have assessed semantic memory (Platel et al., 2003; Di Pietro et al., 2004; Satoh et al., 2006; Plailly et al., 2007), very few have supported the idea of an involvement of the left hemisphere in the musical memory. Our findings globally agree with the literature about the role of left and right hemispheres in musical memory and for first time provide information about distinct musical memory processes that underlies semantic memory, such as recognition and retrieval cognitive processing.

The score-reading task was lateralized in the left hemisphere, while the patient could not remember it in retrieval condition. This IPP task involved syntactic processing. Our result showed a different pattern of musical abilities in hemisphere lateralization. Moreover, the score-reading function was lateralized in the left hemisphere, and its encoding and retrieval took place in the right hemisphere. This left hemisphere lateralization is consistent with a previous study on cortical stimulation during brain mapping (Roux et al., 2007). On the other hand, our data demonstrated that the right hemisphere supported musical episodic memory for these musical abilities. The present study showed great concordance with the Platel study (2005), where the author compare episodic vs. semantic tasks. Platel confirmed right hemisphere dominance for retrieval processes and suggested that musical semantic memory tasks weakly engage the right hemisphere, given that the subtraction of the semantic processes in the episodic tasks did not decrease or remove these activations of the right hemisphere.

The patient's performance in tempo task during the retrieval condition showed that the patient could not recognize which melody was heard on the encoding condition. This result suggests that tempo processing requires interhemispheric contribution. This finding is consistent with a previous study using EEG in musicians (Ma et al., 2012) and found the main effects of tempo at the frontal, temporal, and parietal electrode sites on both the left and the right hemispheres. We assume that the tempo is the most informative code to access episodic memory for music. An interpretation of this data is the notion that after tempo violations (fast and slow melodies), musicians need to integrate newly incoming information to update their mental representation, and create a coherent and semantic melody. Our relevant contribution has demonstrated the role of both hemispheres to integrate the tempo violations during encoding processing.

It is of interest to note that using 3 stimuli for each injection during an IPP are enough to assess the contribution of each hemisphere to music recall. These musical stimuli provide significant data as a significant indicator of a postoperative decline in episodic, syntactic, and semantic memory in TLE patients. Although lateralization of musical functions has been studied by various means (Bogen and Gordon, 1971; Gordon and Bogen, 1974; Plenger et al., 1996), investigations of musical functions on epileptic patients have not been reported yet employing an IPP. Therefore, this study offers an opportunity to analyze the reorganization of musical functions.

In addition, language and memory functions during inactivation of one hemisphere or the other were assessed in this study. We found language dominant-left hemisphere. The patient did not showed specialization of either hemisphere for processing verbal material for memory, while right hemisphere was specialized on visual memory processing. Interestingly, results from verbal memory are similar to those obtained on score-reading task. Meanwhile words and sentences items (verbal recognition) were lateralized in the left hemisphere, their encoding and retrieval took place in the right hemisphere. These results could be related with the lateralization of seizure focus. In adults, there is strong evidence that laterality of temporal lobe seizure focus affects the modality for which memory is most impacted, with verbal memory being most impacted by a left-lateralized lesion and non-verbal (visual) memory most influenced by a right-lateralized lesion (Hermann et al., 1997; Baxendale, 1998; Gleissner et al., 1998; Saling, 2009). Hence, this single case in whom lateralization of verbal memory and musical functions deviates from the expected pattern, showed apparent interhemispheric reorganization of material-specific verbal memory and musical functions. These findings are consistent with the notion that there is neural plasticity in epileptic patients after musical training.

Of particular interest are the advantages of the intracarotid propol procedure. In fact, no adverse symptoms were recorded in the patient during intracarotid injection of propofol, the propofol acts almost instantaneously to depress hemispheric functions unilaterally for a period of 10 min (aprox.) with low doses, meanwhile other drugs as amobarbital acts in shorter periods with higher doses (Gordon and Bogen, 1974; Glosser et al., 1995). This advantage on recovery time allowed us to apply an exhaustive procedure to assess music, language, and memory. On the other hand, other advantages of the IPP are related to its well-established and valid measure of hemispheric dominance. In comparison with the most widely explored potential alternative to the IPP, that is fMRI, the IPP mimics temporary lesion meanwhile fMRI is often used to infer hemispheric dominance for a brain function. Furthermore, EEG recordings from IPP offer information about functional connectivity that is probably essential for optimal brain functioning (Douw et al., 2009). Up to now, the precise effects of lesions or suppression of circumscribed brain areas on functional connectivity in the rest of the brain need further investigations.

On the basis of the present IPP study and pre-surgical results, we attempted to define functional anatomy of episodic and semantic memory for music, besides language, and memory lateralization. According to the available literature, our results demonstrate an overlap between many music-related functions and language processing (Platel et al., 1997; Maess et al., 2001; Koelsch et al., 2002, 2005a,b, 2006; Patel, 2003). Both abilities share fundamental characteristics such as syntactic and semantic structure, and written representations (Patel et al., 1998; Patel, 2003; Koelsch et al., 2004, 2005a,b, 2006). Further arguments in favor of this interpretation are reported by Platel (2005). This author indicated that anterior temporal cortex (mainly of the left hemisphere) would appear particularly involved in semantic memory for musical material. For these reasons, a SAH was selected as surgical technique. The SAH employed in our patient, has been advocated as a less-invasive surgical procedure in order to preserve post-operative cerebral functions. There is increasing evidence that more restricted or selective surgical approaches can help to reduce the cognitive sequelae of surgery as opposed to extended standard ATLR (Katz et al., 1989; Wyler et al., 1995; Helmstaedter and Elger, 1996; Helmstaedter et al., 1996; Pauli et al., 1999; Hori et al., 2003; Alpherts et al., 2006; Paglioli et al., 2006; Helmstaedter et al., 2008). This surgical option, transylvian SAH, resulted in an improvement in post-operative cognitive functions, mainly in verbal memory. Moreover, our case report showed excellent results with regard to seizure outcome and post- operative professional outcome.

Neuropsychological longitudinal results paint a largely positive picture. In pre-operative memory performance the patient showed alterations in encoding and retrieval function. On the other hand, excellent post-operative results in semantic memory were found. It is possible that the mentioned change between pre-operative and post- operative memory could reveal the cognitive consequence of removing lesional vs. non-lesional tissue in temporal lobe surgery. These findings are consistent with the notion that the memory stability is detected 2 years after surgery (Alpherts et al., 2006; Andersson-Roswall et al., 2010). Regarding postoperative results, our data support the claim that the anterior temporal lobe is a crucial component for semantic memory. The handful of studies that have probed semantic processing found indications that semantic memory may be disrupted in TLE patients (Wilkins and Moscovitch, 1978; Glosser et al., 2003; Antonucci et al., 2008). Nevertheless, accumulating evidences are needed to explore the role of anterior temporal lobe in semantic memory processing. Up to now, current literature has a shortage of information on the status of semantic processing in patients with TLE with or without resection.

Neuropathologic analysis following surgical treatment of intractable seizures, revealed massive loss of hippocampal pyramidal neurons of the four sectors of Ammon's horn (CA1–CA4) and discrete reactive astrocytosis. These structural disturbances are considered the most common pathological findings in TLE, accompanied by architectural disturbances of the dentate gyrus. These have been found in epileptic patients (Houser, 1990; Mathern et al., 1997; El Bahh et al., 1999), however, there is no information available regarding the extent to which these structural abnormalities contribute to hippocampal seizure susceptibility or mnesic dysfunction. This is an important prerequisite for further addressing the pathogenic origin and functional impact on TLE.

Overall, the investigation presented in this article convincingly shows the value of the epileptic musician's brain as a model of neuroplasticity, setting the stage for further research.

Our data indicate that musical expertise critically modifies cognitive processes and induces structural and functional interhemispheric plasticity. We agree with Schultz et al. (2005) that it is essential to initiate a database of cases of epilepsy surgery in this special group of patients that integrates the spectrum of musical skills as well as different etiologies and localizations of focal epilepsies.

For the first time our study has identified functional hemispheric lateralization of music, language, and memory in a musician during IPP. Furthermore, current results highlight the efficacy of IPP to achieve a better understanding of the neural basis of music processing and neuroplastic changes. We think that this study, because of the analysis of a single case, does not allow us to reach unequivocal conclusions about this issue. Our IPP did not allow intrahemispheric exploration of all the neural structures possibly involved in musical, memory, and language tasks. These limitations must be taken into account when analyzing the results of neuropsychological studies. In practice, these results, when interpreted with the evidence from the literature, lead us to suggest that clinicians should use a battery for evaluation of musical lateralization in musicians who undergo epilepsy surgery.

In summary, we have confirmed that there is a functional hemispheric lateralization of musical, language, and memory processing in an epileptic professional musician. We have demonstrated that findings on IPP could successfully suggest who might be at risk for postoperative cognitive functional deficits after epilepsy surgery.

Further studies are required to explore factors as musical expertise and epilepsy that critically modifies long-term memory processes and induces brain structural and functional plasticity.

### Conflict of interest statement

The authors declare that the research was conducted in the absence of any commercial or financial relationships that could be construed as a potential conflict of interest.
